# Effect of fluvoxamine on plasma interleukin-6 in patients with major depressive disorder: a prospective follow-up study

**DOI:** 10.3389/fpsyt.2023.1163754

**Published:** 2023-05-25

**Authors:** Xueqin Li, Danfeng Yan, Mei Liao, Li Zhang, ZeXuan Li, Bangshan Liu, Yanjun Chen, Yan Zhang, Jin Liu, LingJiang Li

**Affiliations:** ^1^Department of Psychiatry, National Clinical Research Center for Mental Disorders, and National Center for Mental Disorders, The Second Xiangya Hospital of Central South University, Changsha, Hunan, China; ^2^Mental Health Institute of Central South University, China National Clinical Research Center on Mental Disorders (Xiangya), China National Technology Institute on Mental Disorders, Hunan Technology Institute of Psychiatry, Hunan Key Laboratory of Psychiatry and Mental Health, Changsha, Hunan, China

**Keywords:** major depressive disorder, fluvoxamine, interleukin-6, antidepressant, prospective follow-up study

## Abstract

**Introduction:**

Major depressive disorder (MDD) is associated with low-grade inflammation, and anti-inflammatory treatment can help improve depressive symptoms. A recent study found that fluvoxamine (FLV) can reduce Interleukin-6 (IL-6) production via sigma-1 receptor in inflammation models. However, the anti- IL-6 effect of FLV in treating patients with MDD and whether it can contribute to antidepressant effects remain unclear.

**Methods:**

A total of 65 patients with MDD and 34 healthy controls were recruited at baseline, and 50 patients completed the FLV treatment for 2 months. We assessed depression and anhedonia and collected plasma IL-6 levels at baseline, 1 month, and 2 months after baseline. This study evaluated the changes in clinical measures and IL-6 during treatment and analyzed their association. Further subgroup analyses were conducted in patients with MDD with high, medium, or low IL-6.

**Results:**

Depression and anhedonia were significantly improved in patients with MDD, while the IL-6 did not significantly change after the FLV treatment. However, IL-6 significantly declined after the FLV treatment among patients with MDD with higher baseline IL-6. No significant associations were found between the changes in depressive symptoms and IL-6.

**Conclusion:**

Our study provided preliminary evidence suggesting that the anti-IL-6 effect of FLV might not play a vital role in its antidepressant treatment, at least in patients with MDD with low inflammation. However, for patients with MDD with higher IL-6, FLV can help reduce IL-6 significantly in the antidepressant treatment, which may help guide the individual treatment of MDD with higher IL-6 levels.

**Clinical trial registration:**

https://clinicaltrials.gov/ct2/show/NCT04160377, identifier NCT04160377.

## 1. Introduction

Major depressive disorder (MDD) is associated with high prevalence, low remission rate, and proneness to relapse, leading to a heavy disease burden (1–3). At present, selective serotonin reuptake inhibitors (SSRIs) are still the first-line recommended antidepressant for MDD in several guidelines (4–6). However, the weakness of SSRIs are found common: (1) In approximately 30% of the depressive episodes, MMD patients are not well responsive to adequate dosage of any of the first-line antidepressants, and around 1/3 of the patients developed treatment resistance for antidepressants (4, 7); (2) It usually needs at least 1–2 weeks for the antidepressant to take effect, which might not be effective for patients with a suicidal Ideation (8); (3) Common adverse events might lead to a higher risk of treatment discontinuation (9). Therefore, new pharmacological mechanisms must be identified to improve patient response to antidepressants.

In recent years, preclinical, clinical, and epidemiological evidence has been accumulating regarding the link between inflammation and depression (10, 11). Significant differences in inflammatory factors between patients with MDD and matched healthy controls (HCs) in the central nervous and peripheral blood systems have been found (12, 13), antidepressant could decrease the elevated inflammatory factors in patients with MDD (14), and anti-inflammatory treatment could help improve the patients’ depressive symptoms (15–17). Interleukin-6 (IL-6) is one of the inflammatory markers most closely and consistently related to depression (18). Several studies reported that IL-6 in blood and cerebrospinal fluid (CSF) were significantly higher in patients with MDD (12, 19, 20). In longitudinal analyses, higher IL-6 predicted subsequent chronic course of depression and is more likely to be a risk factor for depression (21–24). SSRIs treatment was associated with decreased IL-6 (14), and anti-IL-6 antibodies were associated with significant improvement in depressive symptoms, especially anhedonia (16, 25, 26). Various pieces of evidence suggest that IL-6 and anhedonia are closely related. Patients with MDD with anhedonia showed higher IL-6 (27), and anti-IL-6 drugs help improve anhedonia in patients with MDD (11, 28). Despite this, only around 30% of patients with MDD showed signs of inflammation, and the levels of inflammation greatly differ (11).

Fluvoxamine (FLV) is a first-line SSRI effective as other SSRIs in treating MDD (5, 6, 8, 29). Recently, a novel anti-inflammatory mechanism of FLV has been identified. In 2019, Rosen et al. first found that FLV could influence the level of IL-6 in mice and human cells in a sigma receptor (S1R)-dependent manner. Similar results were found in replicated studies (30, 31). Moreover, a clinical study attributed the efficiency of FLV on Coronavirus disease 2019 (COVID-19) to its anti-IL-6 mechanism (32).

In summary, the anti-IL-6 mechanism of FLV has been reported in studies on inflammation or viral infection. However, there has been little evidence about the role of anti-IL-6 effect of FLV in treating depression. To explore in which the anti-IL-6 mechanism of FLV perform in patients with MDD, we hypothesized that: (1) the anti-IL-6 mechanism of FLV could contribute to its antidepressant effects. (2) considering the great difference in the levels of IL-6 among MDD subjects and the anti-IL-6 of FLV were confirmed in inflammatory models, patients with higher IL-6 may show a more significant IL-6 decline after the FLV treatment. Using a prospective follow-up design, we aim to explore the anti-IL-6 effect of FLV in treating patients with MDD and whether it helps improve their depressive symptoms.

## 2. Materials and methods

### 2.1. Participants

Sixty-five patients with MDD and 34 healthy controls were recruited. All patients were recruited from the psychiatric outpatient clinic of the Second Xiangya Hospital of Central South University from 2019 to 2021. The patient’s inclusion criteria were: (1) aged between 18 and 55 years and Han Chinese, (2) diagnosed with MDD according to the Mini International Neuropsychiatric Interview (MINI), and met the criteria for a current major depressive episode according to the Statistical Manual of Mental Disorders, fifth edition (DSM-5), (3) had a 17-item Hamilton Rating Scale for Depression (HAMD-17) score of ≥17, (4) had not taken any antidepressant for at least 1 month, and (5) provided written informed consent prior to all procedures in the study. The patient’s exclusion criteria were: (1) with a medical or neurological condition that could affect brain function or with diagnoses of major physical diseases or endocrine diseases, (2) with a lifetime diagnosis of any primary psychiatric disorder other than an MDD, as defined by DSM-5, (3) having an immediate family member with current or a history of bipolar disorder or mania, (4) with current alcohol or other substance abuse disorder, (5) pregnant (or planning for pregnancy) or breastfeeding, (6) had received electroconvulsive therapy in the past 6 months, and (7) with inflammatory disorders or use of any anti-inflammatory drugs (such as antibiotics and non-steroidal anti-inflammatory drugs) in the month before the study.

The medical ethics committee of The Second Xiangya Hospital of Central South University approved this study, and the Ethics approval number is 18630001 (Registration number, ClinicalTrail.gov ID: NCT04160377). Throughout the study, all participants provided written informed consent.

### 2.2. Clinical evaluation

The subjects’ demographic characteristics: age, gender, body mass index (BMI), and clinical characteristics: age at onset, number of previous episodes, total illness duration, and current illness duration, were recorded. The severity of depression was assessed using the 17-item Hamilton Depression Rating Scale (HAMD) (33). Patients with a reduction of ≥50% in baseline HAMD score were regarded as responsive, and those with an exit score of ≤7 on HAMD were regarded as remitted (34, 35). The symptom of anhedonia was assessed using the 14-item self-administered Snaith-Hamilton Pleasure Scale (SHAPS) (36). Finally, the severity of anxiety was assessed using the Hamilton Rating Scale for Anxiety (HAMA) (37). The subjects received assessments with HAMD and SHAPS at the baseline, 1 month, and 2 months after the treatment. HAMA was assessed at the baseline.

### 2.3. Study design and procedure

All patients with MDD receive FLV monotherapy only during the two-month treatment, and there are no adjuvant treatments other than the FLV. Their dosage started at 50 mg/day and was increased to 100 mg/day within 3 days, with the maximum dosage being 300 mg/day. The dosages could be adjusted based on the severity of symptoms, clinical responses, and side effects. The clinical evaluation and whole blood collection were performed before treatment, 1 month, and 2 months after the treatment started.

### 2.4. Plasma cytokine assessment

The fasting whole blood samples (10 mL) were collected in the morning (9–11 am) and placed into EDTA tubes. The tubes were immediately centrifuged (at 3000 r/min for 15 min) at 4°C; then, the plasma was divided into multiple aliquots and frozen in a − 80°C freezer until assay. The plasma level of IL-6 was examined by a high sensitivity enzyme-linked immunosorbent assay (ELISA, Quantikine HS Human IL-6, R&D Systems, Minneapolis, Minnesota) according to the manufacturer’s protocol. The assay has a lower limit of quantitation of 0.2 pg./mL, a range of 0.2–10 pg./mL, and a sensitivity of 0.09 pg./mL. All samples were tested in duplicate. The experimental operators were blinded to all the clinical data.

### 2.5. Statistics

The normality of the variables was tested using the Kolmogorov–Smirnov test. The skewed data were transformed by log or square root to achieve normal distribution. Continuous variables were presented using mean and standard deviation (SD), and categorical variables were presented using percentages. IL-6 values were converted to standardized Z-scores in HC and MDD groups, and samples with an absolute Z-score > two were removed (38).

Firstly, demographic information and clinical characteristics (Age, BMI, HAMD scores at baseline, IL-6 at baseline) between the MDD and HC groups were compared by independent samples *t*-tests and Chi-square tests. Secondly, a repeated-measures analysis of variance (ANOVA) was used to evaluate the changes in HAMD, SHAPS scores, and IL-6 levels during treatment. The correlation between the changes in any two of the HAMD score, the SHAPS score, and IL-6 were tested using partial correlational analysis, controlling for age, sex, and BMI. Bonferroni correction was used for multiple tests. Thirdly, hierarchical linear regression was used to assess the impact of baseline IL-6 on the changes in the HAMD score, and hierarchical logistic regression was used to assess the influence of baseline IL-6 on the response rate, controlling for age, sex, and BMI. Moreover, hierarchical linear regression was performed to examine the impact of baseline IL-6 on the changes in IL-6 during treatment, controlling for age, gender, weight, and HAMD.

Finally, referring to other studies, this study divides patients with MDD into three subgroups (top, middle, and bottom one-third) from high to low based on the distribution of baseline IL-6 levels (22, 39). Then, *t*-tests were used to compare the three subgroups with HCs separately and make the minimum subject adjustment to ensure that the subgroups are significantly higher, medium, and lower than HCs statistically. The change in IL-6 and the association between IL-6 and depressive symptoms of the three subgroups were further tested using the analyses described above (*T*-tests, ANOVA, multiple regression).

The significance level was set at *p* < 0.05 (two-tailed). The Statistical Package for the Social Sciences (SPSS), version 26 for Windows (SPPS Inc., Chicago, IL), was used to create the database and perform the statistical analyses.

## 3. Results

### 3.1. Demographic and clinical characteristics

Among the 65 patients recruited, 50 completed the two-month FLV treatment and all the assessments at three time points, three developed bipolar disorder, and 12 dropped out for personal reasons or COVID-19. Table 1 shows the detailed demographic and clinical characteristics of patients with MDD and HCs. There was no significant difference regarding age, gender, BMI, or baseline IL-6 between MDDs and HCs.

**Table 1:** Demographic and clinical characteristics.

	MDD (*n* = 59)	HC (*n* = 34)	*p*
*Demographic characteristics*			
Age (year)	25.86 ± 5.27	24.47 ± 4.07	0.158
BMI (kg/m^2^)	20.91 ± 2.56	20.55 ± 2.92	0.528
Gender (male/female)	17/42	9/25	0.811
*Scores of symptoms*			
HAMD (baseline)	22.28 ± 4.13	0.91 ± 1.29	**<0.001**
*Clinical characteristics*			
Age of onset (year)	23.63 ± 5.20	–	–
First episode/recurrence	29/33	–	–
Previous episodes (n)	1.87 ± 1.08	–	–
Total illness duration (month)	27.19 ± 30.22	–	–
Current illness duration (month)	9.59 ± 14.55	–	–
Baseline IL-6 value	1.091 ± 0.648	1.195±0.757	0.394

BMI, body mass index; HAMD, Hamilton Depression Rating Scale; HAMA, Hamilton Rating Scale for Anxiety. **PS**: Data in the above table are all baseline data.

### 3.2. IL-6 changes during treatment in patients with MDD and subgroups

**Figure 1:**
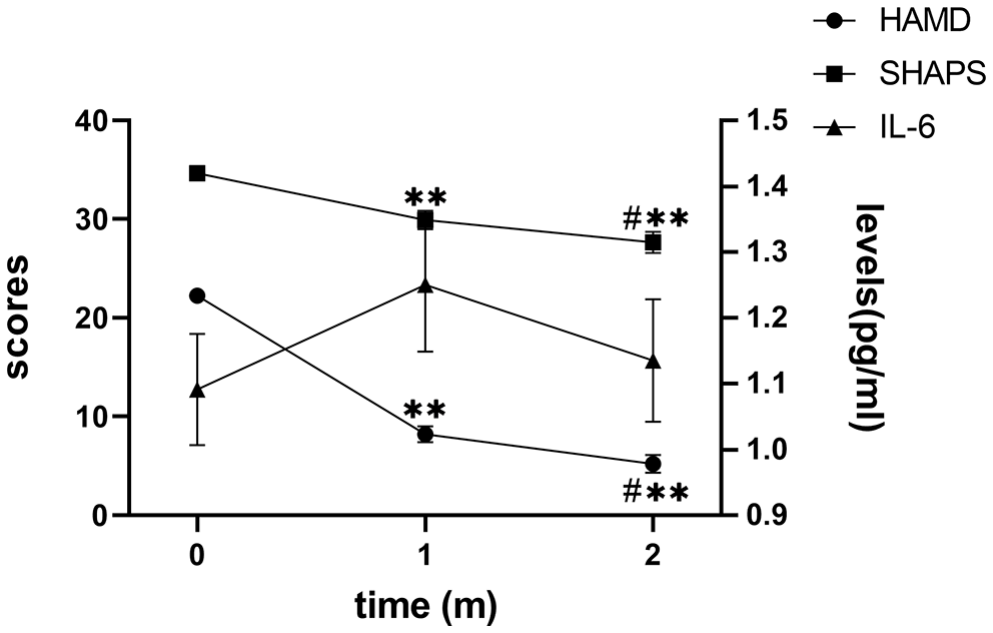
The trend of change in the HAMD score, SHAPS score, and IL-6 level. HAMD, Hamilton Depression Rating Scale; SHAPS, Snaith-Hamilton Pleasure Scale; IL-6, Interleukin 6; m, month. The Y axis on the right indicates IL-6 levels; the Y axis on the left indicates the HAMD or SHAPS scores. Mark * means *p* < 0.05 vs. baseline IL-6; Mark ** means *p* < 0.01 vs. baseline IL-6; Mark # means *p* < 0.05 when 1 m vs. 2 m.

**Figure 2:**
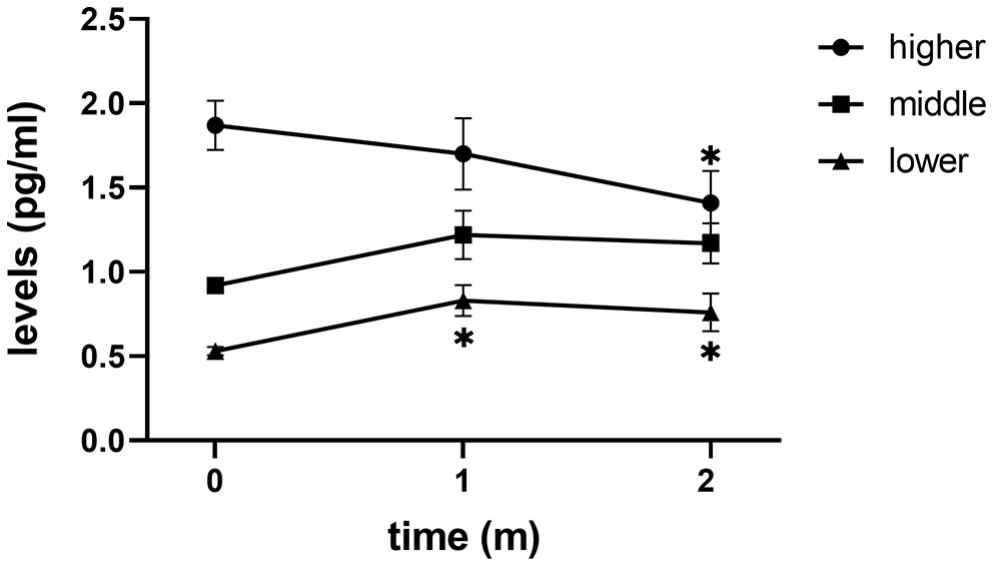
The trend of change in IL-6 levels of three subgroups. m, month; Mark * means *p* < 0.05 vs. baseline IL-6.

After receiving treatment with FLV, the overall depressive symptoms and anhedonia improved significantly, and IL-6 did not change significantly in patients with MDD (Figure 1). However, in the subgroup analysis, we discovered that IL-6 declined significantly after receiving FLV treatment among patients with a higher baseline IL-6. Moreover, this significant change was unique to patients with a higher baseline IL-6; the lower or medium subgroup did not show that (Figure 2).

### 3.3. Correlations between IL-6 and the clinical measures

No significant correlation was found between IL-6 and HAMD or SHAPS changes in patients with MDD or subgroups; only a weak correlation was found between HAMD and SHAPS changes during the second month of the treatment (Table 2).

**Table 2:** Correlation between the changes of HAMD, SHAPS, and IL-6.

Change duration	HAMD & IL-6		SHAPS & IL-6		HAMD & SHAPS	
	*r*	*p*	*r*	*p*	*r*	*p*
1st month	−0.151	0.317	0.082	0.590	0.108	0.450
2nd month	0.043	0.786	0.101	0.518	**0.451**	**0.001** ^**a**^
Two months of treatment	0.166	0.275	−0.077	0.617	0.305	0.033

IL-6, Interleukin 6; HAMD, Hamilton Depression Rating Scale; SHAPS, Snaith-Hamilton Pleasure Scale. ^a^ Significant association (with Bonferroni corrections for multiple tests: *p* < 0.016).

Regarding the responsive group (RG) and the unresponsive group (URG), there was no significant difference in IL-6 between RG and URG after one or 2 months of treatment. In addition, no significant correlation between the changes in HAMD scores and IL-6 was found in the RG or the URG, indicating that IL-6 was not significantly associated with the HAMD scores in the FLV treatment, regardless of the patient’s treatment responsiveness (Supplementary Table S1).

Finally, no significant association was found between baseline IL-6 and the improvement of depressive symptoms in the patients with MDD or the subgroups, indicating that the baseline IL-6 could not predict the anti-depressant effect of FLV in our study (Supplementary Table S2).

## 4. Discussion

To our knowledge, this study is the first longitudinal study to formally explore the changes and significance of IL-6 in FLV antidepressant treatment, especially in patients with MDD without comorbidity in inflammatory disorders. We found that the baseline IL-6 of the patients was significantly related to their post-treatment level of IL-6. Our results especially showed that higher baseline IL-6 predicted a significant decline of IL-6 after the FLV treatment. The results suggest that FLV may play a role in reducing the inflammation in patients with MDD with higher levels of inflammation. It may inform individual treatment for patients with MDD with higher levels of inflammation.

There has yet to be a consensus on the changes in IL-6 levels in SSRI treatments. High heterogeneity across studies limits the credibility and interpretation of the findings in the meta-analysis (14, 40). Among all the SSRI studies, FLV is shown only in a Japanese MDD study (41). However, the contradictions in that study are that the IL-6 level in patients with MDD was too much higher than that in most studies, both before and after treatment (week 0: 134 ± 53 pg./mL; week 8: 106 ± 34 pg./mL), and it failed to match the IL-6 ELISA kit range which is 0.1 to 10 pg./mL. On the other hand, the IL-6 levels in our study were consistent with most previous studies (22, 42–44), and IL-6 did not significantly change in either 1 or 2 months.

Considering the great difference between the patients with MDD in IL-6, and only around 30% of patients with MDD showed signs of inflammation, the study divided the patients into higher, medium, and lower subgroups based on their baseline IL-6. We did find that the patients in the higher subgroup had a more significant decline in IL-6 during FLV treatment. The medium or lower subgroup did not show the same decline. This result suggests that patients with MDD with a higher baseline IL-6 may have more IL-6 reduction when receiving FLV treatment. Here, we have a cautious inference about the possible biological mechanism behind this result. Firstly, Rosen’s study confirmed that FLV could reduce IL-6 in an S1R-dependent manner, found that S1R is uniquely poised to control inositol-requiring enzyme 1*α*(IRE1) activity sensitively and to affect the expression of active X-box binding protein 1 (XBP1) during inflammation (31). At the same time, other studies reveal that the IRE1-XBP1 pathway can activate IL-6 signaling expression (45). Therefore, we would cautiously infer that FLV might reduce IL-6 through S1R to inhibit the IRE1-XBP1 pathway. For the result that higher IL-6 showed more IL-6 reduction in FLV treatment, we considered that the active IL-6 activity buffer system might play a role. The buffer system inhibits the IL-6 trans-signaling in the healthy state. While in the inflammatory state, IL-6 trans-signaling is activated, further increasing IL-6 transcription (46). Moreover, some studies suggest that adding IL-6 leads to increased activation of the IRE1-XBP1 pathway (47). Hence, patients with MDD with higher IL-6 may have an increased IRE1-XBP1 pathway activation. With the activation of the IRE1-XBP1 pathway, FLV-S1R may further control IRE1 to reduce IL-6 (Supplementary Figure S3). That could be why in our study, FLV did not show a significant anti-IL-6 effect when baseline IL-6 was at a low level, but the effect of FLV on IL-6 became obvious with the increase of baseline IL-6.

In terms of the relationship between IL-6 and clinical measures of depression, no significant correlation was found between depression and IL-6 change; baseline IL-6 could not predict the change in depressive symptoms after the FLV treatment, either in overall subjects or subgroups. In short, IL-6 is not significantly correlated with either overall depressive symptoms or anhedonia. We speculate that these non-significant results may be that the serotonin mechanism plays the dominant role in FLV antidepressant treatment rather than the anti-IL-6 mechanism, at least in patients with MDD without inflammatory disorders.

Overall, one of the most important implications of our findings is to inform the clinical treatment of MDD, especially for patients with higher inflammation. In addition, our findings may inform future research on this topic. Importantly, the results in the current study are preliminary and need to be repeated with larger samples. Hence, future research may recruit a larger sample of patients with MDD and explore whether the change of IL-6 is more obvious in patients with MDD with higher IL-6, especially in patients with comorbidity of inflammatory disorders. Also, future research may wish to go further from our study and see whether when the patient’s baseline IL-6 reaches a certain threshold, the mechanism of FLV-IL-6 can significantly help the antidepressant efficiency. It can also compare the efficacy of FLV with other SSRIs for patients with MDD with different levels of IL-6. Research on these areas may inform the development of antidepressants and may advance individualized treatments for patients with MDD, as well as improvement in the prognosis of patients with MDD.

## 5. Limitation

Some limitations in this study should be noted. Firstly, the patients in the study were relatively young and did not have severe levels of inflammation. Moreover, our study found that Patients with MDD with higher baseline levels of IL-6 experience a more significant IL-6 decline during the FLV treatment. Future studies may therefore include patients with MDD with higher levels of inflammation on average and patients of multiple ages to determine the precise significance of the FLV-IL-6 pathway in treating MDD. Secondly, our findings were preliminary. The sample size was relatively small, which might limit the statistical power of the subgroup analysis. In summary, future research requires larger sample sizes and patients with MDD with different levels of IL-6, including significantly higher levels.

## 6. Conclusion

In summary, this study is the first longitudinal study to explore the changes and the significance of IL-6 among patients with MDD during FLV treatment with multiple time points. Our results suggest that the anti-IL-6 effect of FLV might not play a vital role in its antidepressant treatment, at least in patients with MDD with low inflammation. Moreover, for patients with higher baseline IL-6, FLV treatment resulted in a significant reduction in IL-6 levels, which provided preliminary evidence for the individual treatment of patients with MDD with higher inflammation. Future studies with larger samples of patients with MDD with higher IL-6 are needed, which have significant clinical implications for improving the prognosis of patients with MDD. Additionally, future studies may incorporate other SSRIs that have no affinity with S1R (such as paroxetine) or act as S1R antagonists (like sertraline) (48) to explain the efficacy of S1R-mediated anti-IL-6 in antidepressant treatment more accurately.

## Data availability statement

The original contributions presented in the study are included in the article/Supplementary material, further inquiries can be directed to the corresponding authors.

## Ethics statement

The studies involving human participants were reviewed and approved by the Medical Ethics Committee of The Second Xiangya Hospital of Central South University approved this study. The patients/participants provided their written informed consent to participate in this study.

## Author contributions

XL: conceptualization, data curation, formal analysis, investigation, writing – original draft, and writing – review and editing. DY, ML, LZ, ZL, BL, and YZ: investigation. YC: writing – review and editing. LL: conceptualization, funding acquisition, investigation, methodology, project administration, supervision, and writing – review and editing. JL: conceptualization, funding acquisition, project administration, supervision, and writing – review and editing. All authors contributed to the article and approved the submitted version.

## Funding

This study was supported by the National Key Research and Development Program of China (2019YFA0706200); Natural Science Foundation of China (82171518); Brain Science and Brain-Inspired Intelligence Technology (2021ZD0202002); and the Fundamental Research Funds for the Central Universities of Central South University (1053320211506 to MM). All the Funding Sources above had no further role in study design; in the collection, analysis and interpretation of data; in the writing of the report; and in the decision to submit the paper for publication.

## Conflict of interest

The authors declare that the research was conducted in the absence of any commercial or financial relationships that could be construed as a potential conflict of interest.

## Publisher’s note

All claims expressed in this article are solely those of the authors and do not necessarily represent those of their affiliated organizations, or those of the publisher, the editors and the reviewers. Any product that may be evaluated in this article, or claim that may be made by its manufacturer, is not guaranteed or endorsed by the publisher.
