# Supplementary material for: Effect of fluvoxamine on plasma interleukin-6 in patients with major depressive disorder: a prospective follow-up study
Source: Front Psychiatry. 2023 May 25;14:1163754. doi: 10.3389/fpsyt.2023.1163754 (PMC10247978; doi:10.3389/fpsyt.2023.1163754)
Supplement: Supplementary file 1 [file Table_1.DOCX]

**Supplemental Table S1**

**Table. S1.**the results in the responsive group and the unresponsive group

|  | 1M | *2M* |
| --- | --- | --- |
| response rate | 77.78% | 80.77% |
| remission rate | 55.56%, | 78.85% |
| **Responsive group(RG)** | 1.70±0.87 | 1.24±0.62 |
| IL-6 | 1.501±1.307 pg/ml, | 1.540± 2.220 pg/ml |
| Association of △IL-6 and △HAMD (r, p) | r=-0.071, p=0.671 | r=0.019 p=0.911; |
| **Unresponsive group(URG)** |  |  |
| IL-6 | 0.950±0.613 pg/ml | 1.282±0.447 pg/ml |
| Association of △IL-6 and △HAMD in 1 month (*r, p*) | r=0.030 p=0.944; | r=0.157, p=0.737 |
| The difference of Il-6 between RG and URG | p=0.182 | p=0.247 |

**Supplemental Table S2**

**Table S2. subgroup based on baseline IL-6 value and analysis between three subgroups (t/correlation)**

|  | Higher MDD | *Medium MDD* | Lower MDD |
| --- | --- | --- | --- |
| Baseline IL-6 | 1.87±0.60 | 0.92±0.15 | 0.53±0.10 |
| HC（*p*） | 0.003 | 0.053 | ＜0.001 |
| 1 M IL-6 | 1.70±0.87 | 1.24±0.62 | 0.83±0.41 |
| 2 M IL-6 | 1.41±0.81 | 1.17±0.51 | 0.80±0.45 |
| 1M decline （*p*） | 0.387 | 0.063 | 0.020 |
| 2M decline （*p*） | 0.026 | 0.075 | 0.046 |
| Association of △IL-6 and △HAMD in 1 month (*r, p*) | -0.045, 0.884 | -0.301, 0.296 | -0.102, 0.740 |
| Association of △IL-6 and △HAMD in 2 month (*r, p*) | -0.430, 0.125 | 0.373, 0.209 | 0.640, 0.025 |
| Association of △IL-6 and △SHAPS in 1 month (*r, p*) | 0.519, 0.069 | 0.391, 0.167 | -0.341, 0.254 |
| Association of △IL-6 and △SHAPS in 2 months (*r, p*) | -0.007, 0.982 | -0.018, 0.955 | -0.442, 0.150 |
| Prediction of baseline IL-6 on 1M △ HAMD (*p*) | 0.140 | 0.989 | 0.694 |
| Prediction of baseline IL-6 on 2M △ HAMD (*p*) | 0.650 | 0.840 | 0.495 |
