# Supplementary figures and images for: Effect of fluvoxamine on plasma interleukin-6 in patients with major depressive disorder: a prospective follow-up study
Source: Front Psychiatry. 2023 May 25;14:1163754. doi: 10.3389/fpsyt.2023.1163754 (PMC10247978; doi:10.3389/fpsyt.2023.1163754)

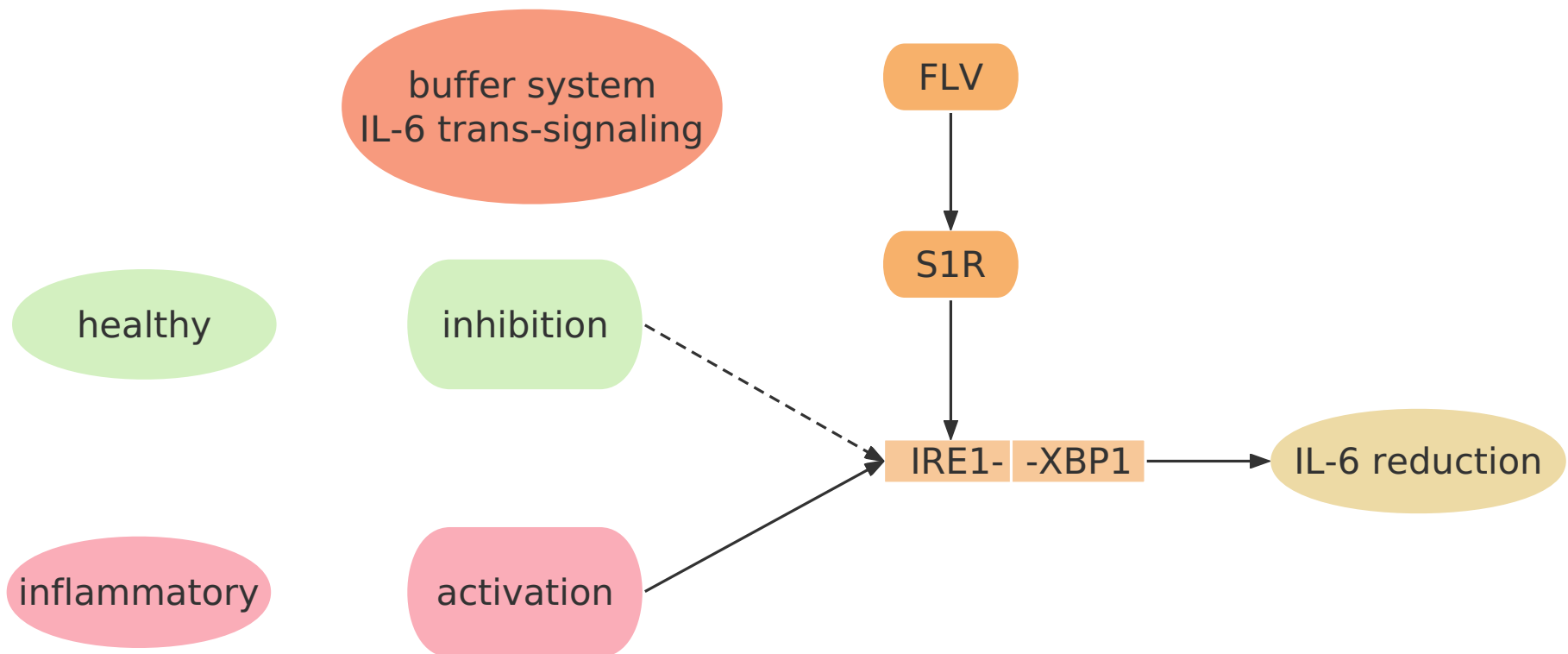

Supplement: Supplementary file 2 [file Data_Sheet_1.PDF]
